# Supplementary material for: Resistance and Aerobic Training Were Effective in Activating Different Markers of the Browning Process in Obesity
Source: Int J Mol Sci. 2023 Dec 24;25(1):275. doi: 10.3390/ijms25010275 (PMC10778972; doi:10.3390/ijms25010275)
Supplement: Supplementary file 1 [file ijms-25-00275-s001.zip › Supplementary File S1.pdf]

SUPPLEMENTAL DIGITAL CONTENT

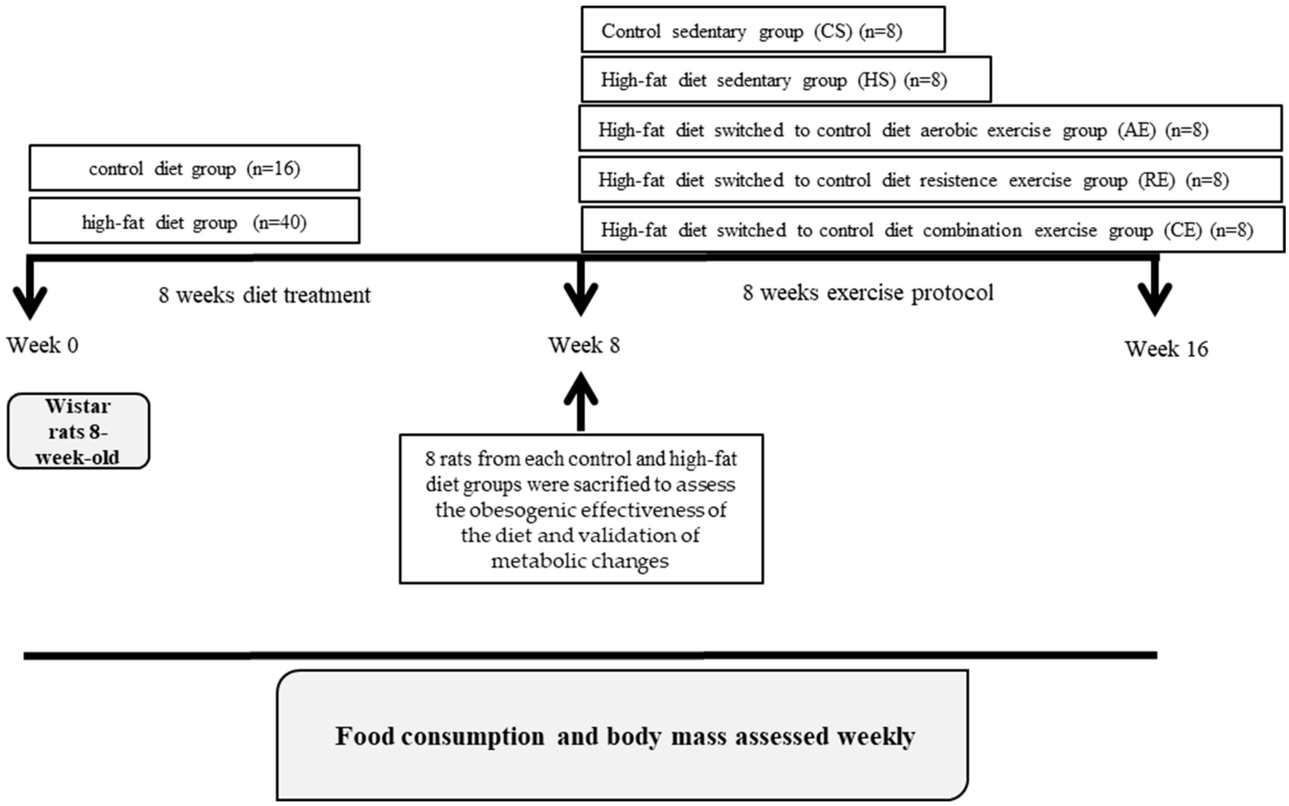

Figure S1. Experimental design

Table S1. Components of the diets enriched with pork lard and soybean oil.

Components/100 g	Lard
Control feed	50
Sucrose	10
Casein	20
Soybean oil	2
Pork lard	18

Table S2. Description of the resistance training protocol

RESISTANCE TRAINING				
Week	Climbing series	Movement repetitions per climbing series	Interval between series (seconds)	Training load (% maximum load)
1st	4–8	8–12	60	1 = 50% /1 = 75% /1 = 90% / 1 = 100% / an additional 30 g until failure
2nd	4–8	8–12	60	1 = 50% /1 = 75% /1 = 90% / 1 = 100% / an additional 30 g until failure
3rd	4–8	8–12	60	1 = 50% /1 = 75% /1 = 90% / 1 = 100% / an additional 30 g until failure

RESISTANCE TRAINING				
Week	Climbing series	Movement repetitions per climbing series	Interval between series (seconds)	Training load (% maximum load)
4th	4–8	8–12	60	1 = 50% /1 = 75% /1 = 90% / 1 = 100% / an additional 30 g until failure
5th	4–8	8–12	60	1 = 50% /1 = 75% /1 = 90% / 1 = 100% / an additional 30 g until failure
6th	4–8	8–12	60	1 = 50% /1 = 75% /1 = 90% / 1 = 100% / an additional 30 g until failure

RESISTANCE TRAINING				
Week	Climbing series	Movement repetitions per climbing series	Interval between series (seconds)	Training load (% maximum load)
7th	4–8	8–12	60	1 = 50% /1 = 75% /1 = 90% / 1 = 100% / an additional 30 g until failure
8th	4–8	8–12	60	1 = 50% /1 = 75% /1 = 90% / 1 = 100% / an additional 30 g until failure

Source: MÔNICO-NETO et al. (2015)

Table S3. Description of the aerobic training protocol

Week	Duration of each training session	Percentage of V_{max}.
1st	30 minutes	30%
2nd	40 minutes	30%
3rd	50 minutes	30%
4th	60 minutes	30%
5th	60 minutes	40%
6th	60 minutes	45%
7th	60 minutes	50%
8th	60 minutes	55%

V_{max}: maximum Velocity.
Source: FERREIRA et al. (2007)

Table S4. Description of the combined training protocol

RESISTANCE TRAINING				
Week	Climbing series	Movement repetitions per climbing series	Interval between series (seconds)	Training load (% maximum load)
1st	3–4	8–12	60	1 = 50% /1 = 75% /1 = 90% / 1 = 100%
2nd	3–4	8–12	60	1 = 50% /1 = 75% /1 = 90% / 1 = 100%
3rd	3–4	8–12	60	1 = 50% /1 = 75% /1 = 90% / 1 = 100%
4th	3–4	8–12	60	1 = 50% /1 = 75% /1 = 90% / 1 = 100%
5th	3–4	8–12	60	1 = 50% /1 = 75% /1 = 90% / 1 = 100%

RESISTANCE TRAINING				
Week	Climbing series	Movement repetitions per climbing series	Interval between series (seconds)	Training load (% maximum load)
6th	3–4	8–12	60	1 = 50% /1 = 75% /1 = 90% / 1 = 100%
7th	3–4	8–12	60	1 = 50% /1 = 75% /1 = 90% / 1 = 100%
8th	3–4	8–12	60	1 = 50% /1 = 75% /1 = 90% / 1 = 100%

Source: Adapted from MÔNICO-NETO et al. (2015)

AEROBIC TRAINING		
Week	Duration of each training session	Percentage of V_{max}
1st	15 minutes	30%
2nd	20 minutes	30%
3rd	25 minutes	30%
4th	30 minutes	30%
5th	30 minutes	40%
6th	30 minutes	45%
7th	30 minutes	50%

AEROBIC TRAINING		
Week	Duration of each training session	Percentage of V_{max}
8th	30 minutes	55%

V_{max}: Maximum Velocity.
Source: Adapted from FERREIRA et al. (2007)

Determination of fat and protein content in the carcasses

The fat content of the carcasses was determined using the gravimetric method described by OLLER DO NASCIMENTO & WILLIAMSON (1988). To that end, the carcasses were previously softened in the autoclave and then homogenized in water at 1:3 (weight: volume). Lipids were extracted from approximately 3-gram aliquots of the homogenate using the method described by STANSBIE et al. (1976). The protein content of the carcasses was determined using the method described by BRADFORD. The results were expressed in g/100g of carcass.

Reference:

Oller do Nascimento CM, Williamson DH. Tissue-specific effects of starvation and refeeding on the disposal of oral [1-14C]triolein in the rat during lactation and on removal of litter. Biochem J. 1988. doi: 10.1042/bj2540539.

Stansbie D, Brownsey RW, Cretaz M & Denton RM (1976) Acute effects in vivo of anti-insulin serum on rates of fatty acid synthesis and activities of acetyl-coenzyme A carboxylase and pyruvate dehydrogenase in liver and epididymal adipose tissue of fed rats. Biochemical Journal 160, 413–416.

Table S5. Body parameters after 8- and 16-week treatment of CS and HS groups

Parameters	CS (n=8) 8 weeks	HS (n=8) 8 weeks	CS (n=8) 16 weeks	HS (n=8) 16 weeks	P
**Relative RET weight (g/100g)[a]**	0.96 ± 0.07	1.93 ± 0.13*	0.93 ± 0.08*	2.50 ± 0.14*#	<0.01
**Relative EPI weight (g/100g)[a]**	0.97 ± 0.08	1.24 ± 0.40	1.35 ± 0.11	2.69 ± 0.24*#	<0.01
**Relative MES weight (g/100g)[a]**	0.77 ± 0.11	1.25 ± 0.01	0.82 ± 0.05	1.74 ± 0.17*#	<0.01
**Relative SUB weight (g/100g)[a]**	0.37 ± 0.05	0.48 ± 0.09	0.60 ± 0.08	0.98 ± 0.16*+	<0.01
**Adiposity (g/100g)[b]**	2.24 (1.77-3.04)	3.69 (3.04-5.30)*	2.93 (2.40-4.24)	7.38 (4.41-8.69)*#	<0.01
**Total carcass protein (g/100g body mass)[a]**	7.05 ± 0.92	9.84 ± 1.06	9.42 ± 0.55	9.50 ± 0.08	0.07
**Total carcass fat (g/100g body mass)[a]**	10.54 ± 0.58	12.36 ± 0.77	10.16 ± 0.73	17.63 ± 2.42*#	<0.01

For parametric measures (a), values are expressed as means ± standard error of the mean (SEM); for non-parametric measures (b), values are expressed as the median (minimum–maximum); *: significant difference of CS 8 weeks group; #: significant difference of CS 16 weeks group; +: significant difference of HS 8 weeks group; EPI – Epididymal adipose tissue; RET – Retroperitoneal adipose tissue; MES – Mesenteric adipose tissue; SUB - Subcutaneous adipose tissue. Adiposity: Σ of epididymal, retroperitoneal and mesenteric relative weight of tissues (g body mass). CS - control sedentary group; HS - high-fat sedentary group.

Table S6. Body parameters after 16-week treatment of AE, RE and CE groups

Parameters	AE (n=8)	RE (n=8)	CE (n=8)	P
**Relative RET weight (g/100g)**	1.0 ± 0.11	1.1 ± 0.12	1.1 ± 0.17	0.88
**Relative EPI weight (g/100g)**	1.3 ± 0.15	1.6 ± 0.14	1.3 ± 0.19	0.44
**Relative MES weight (g/100g)**	0.6 ± 0.06	0.8 ± 0.08	0.7 ± 0.11	0.23
**Relative SUB weight (g/100g)**	0.5 ± 0.08	0.5 ± 0.08	0.5 ± 0.09	0.88
**Adiposity (g/100g)**	3.0 ± 0.32	3.8 ± 0.44	3.3 ± 0.46	0.37
**Total carcass protein (g/100g body mass)**	10.4 ± 1.51	10.4 ± 0.71	12.4 ± 0.66	0.25
**Total carcass fat (g/100g body mass)**	7.9 ± 0.87	8.3 ± 0.61	7.2 ± 0.53	0.51

The values are expressed as means ± standard error of the mean (SEM); EPI – Epididymal adipose tissue; RET – Retroperitoneal adipose tissue; MES – Mesenteric adipose tissue; SUB - Subcutaneous adipose tissue. Adiposity: Σ of epididymal, retroperitoneal and mesenteric relative weight of tissues (g body mass). AE - aerobic exercise, RE - resisted exercise and CE - combined exercise group.

Table S7. Body mass: initial, after 8 weeks and after 16-weeks treatment of CS, HS, AE, RE and CE groups

Body mass (g)	CS	HS	AE	RE	CE	P
**Initial**	294.6 ± 6.7	284 ± 4.9	291.3 ± 6.7	292 ± 4.2	300.8 ± 7.6	0.44
**After 8 weeks**	409.4 ± 13.1	452.4 ± 6.2	471.8 ± 18.7*	463.5 ± 10.1	472.8 ± 16.4*	< 0.01
**After 16 weeks**	464.6 ± 14.4	529.5 ± 7.9*	468.9 ± 16.5#	473.3 ± 13.2#	479.6 ± 15.2	< 0.01

The values are expressed as means ± standard error of the mean (SEM); CS - control sedentary group; HS - high-fat sedentary group; AE - aerobic exercise, RE - resisted exercise and CE - combined exercise group. Eight animals per group. *: significant difference of CS. #: significant difference of HS.

Assessment of biochemical parameters

The biochemical parameters were analyzed using the serum samples collected on the day of the euthanasia. Triglycerides, total cholesterol, high-density lipoprotein (HDL), and glucose were measured using a laboratory kit provided by Labtest Diagnóstica S.A. (Lagoa Santa, State of Minas Gerais, Brazil). The tests were performed on 96-well plates, and the absorbance of the samples was measured using an Epoch™ spectrophotometer (Agilent Technologies, Winooski, VT, EUA) for the appropriate wavelength for each test, according to the manufacturer's instructions.

Table S8. **Total cholesterol, Glucose, Triglycerides and HDL after 8- and 16-week treatment of CS and HS groups**

Parameters	CS 8 weeks	HS 8 weeks	CS 16 weeks	HS 16 weeks	P
**Total cholesterol (mg/ dL)[a]**	105.0 ± 5.28	118.0 ± 6.69	114.0 ± 5.00	132.2 ± 6.40*	0.02
**Glucose (mg/ dL)[a]**	119.9 ± 3.90	129.5 ± 3.35	117.0 ± 4.73	137.8 ± 6.84#	0.02
**Triglycerides (mg/ dL)[b]**	105.5 (92.9-113.5)	109.5 (94.7-136.0)	101.4 (82.4-138.5)	101.0 (91.2-158.5)	0.59
**HDL (mg/dL)[a]**	51.8 ± 3.05	46.2 ± 4.02	49.0 ± 3.46	58.1 ± 3.79	0.14

For parametric measures (a), values are expressed as means ± standard error of the mean (SEM); for non-parametric measures (b), values are expressed as the median (minimum–maximum); *: significant difference of CS 8 weeks group and #: significant difference of CS 16 weeks group. HDL - High-Density Lipoproteins. CS - control sedentary group; HS - high-fat sedentary groups. Samples vary from six to seven animals.

Table S9. **Total cholesterol, Glucose, Triglycerides and HDL after 16-week treatment of AE, RE and CE groups**

Parameters	AE (n=8)	RE (n=8)	CE (n=8)	P
**Total cholesterol (mg/ dL)**	114.3 ± 9.30	126.7 ± 9.00	119.4 ± 6.12	0.57
**Glucose (mg/ dL)**	116.2 ± 1.80	121.9 ± 3.74	123.9 ± 4.29	0.19
**Triglycerides (mg/ dL)**	102.2 ± 3.53	110.0 ± 4.78	102.8 ± 2.71	0.28
**HDL (mg/dL)**	49.8 ± 1.42	59.8 ± 5.66	56.0 ± 3.48	0.21

For parametric measures, values are expressed as means ± standard error of the mean (SEM). HDL - High-Density Lipoproteins. AE - aerobic exercise, RE - resisted exercise and CE - combined exercise groups.

A)

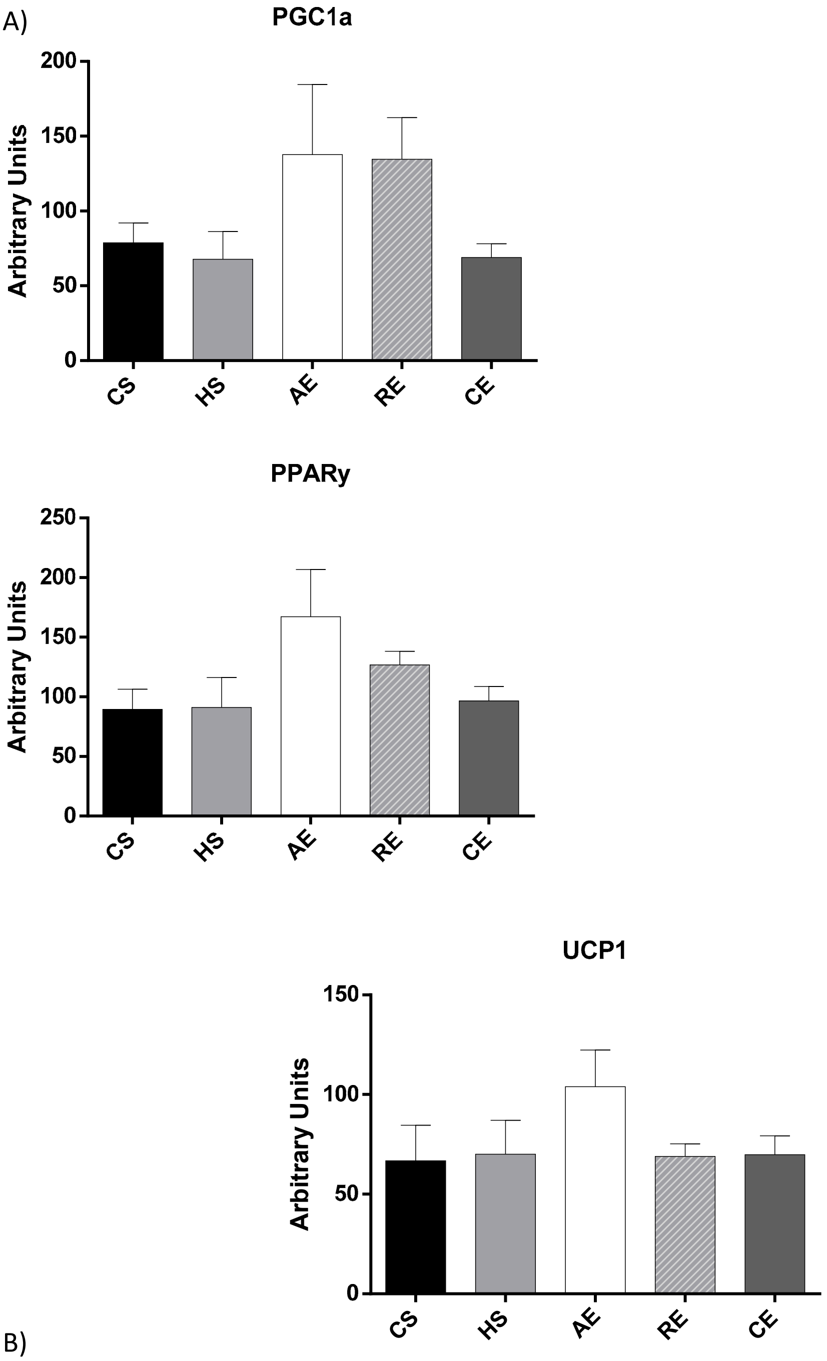

B)

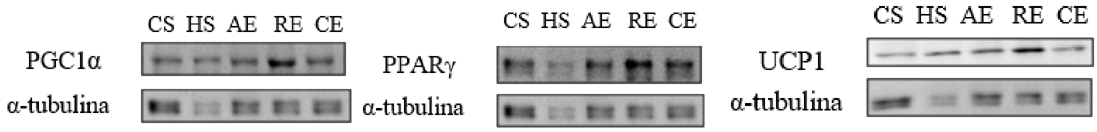

Figure S2. Retroperitoneal adipose tissue protein content by Western blotting from PGC1a, PPARy and UCP1 of CS, HS, AE, RE and CE groups (**A**) and intensity of each band of the proteins analyzed and respective housekeeping protein (β-actin) (**B**). Samples vary from four to seven animals.
