# Supplementary material for: Resistance and Aerobic Training Were Effective in Activating Different Markers of the Browning Process in Obesity
Source: Int J Mol Sci. 2023 Dec 24;25(1):275. doi: 10.3390/ijms25010275 (PMC10778972; doi:10.3390/ijms25010275)
Supplement: Supplementary file 1 [file ijms-25-00275-s001.zip › Supplementary File S2-Individual data.pdf]

+Individual data (manuscript)				
**Reference - FIGURE 1**				
**Adiposity (g/100g)**				
**CS**	**HS**	**AE**	**RE**	**CE**
2.939116	6.730815	1.919947	3.588460	5.497984
2.947404	7.930396	1.941654	3.555553	2.353723
2.900901	7.656498	2.440429	3.314105	1.710892
3.481206	7.805010	3.981702	2.603161	1.974469
2.938800	5.228406	4.393082	3.221769	3.883486
2.401823	8.695893	3.058468	4.588914	2.879653
	7.106475	2.758796		3.664302
		3.608816		4.590161
**Weight delta (grams)**				
133	235	101	159	183
147	217	112	170	165
186	256	192	150	121
163	231	177	110	173
146	208	192	159	170
178	212	169	195	124
107	278	125	171	165
	280	189		201
		145		
**Total carcass fat (g/100g)**				
8.01880	20.39179	6.51147	8.34083	8.11499
11.64421	21.74861	9.18428	10.83676	5.67828
11.66592	17.90088	5.96716	7.58588	7.36573
8.95375	24.69560	10.66671	8.48707	6.26455
12.00006	11.01054	7.32521	6.18199	13.51245
8.69925			11.76056	6.73556
			8.49867	9.29976
**Cumulative Caloric Intake Total (kcal) (Weeks)**				
3728.13	3294.0	4325.19	3451.74	4341.37
3871.63	3290.4	4415.77	3652.31	4292.84
4152.89	3848.4	4312.25	4150.50	3904.64
3840.06	3837.6	4150.50	4076.10	4001.69
		3827.00		

Individual data (manuscript)	
Reference - FIGURE 2	
Final load (grams)	
**RE**	**CE**
1122	730
1080	562
1045	864
1004	825
1672	676
1434	960
1233	860
1549	854
	898

	Strength training progression (grams)																
	**RE**								**CE**								
1	560	630	683	615	688	684	573	649	400	382	324	465	406	360	380	394	538
2	740	840	773	705	988	894	813	949	400	382	324	465	406	360	380	494	538
3	800	900	803	825	1258	1104	843	1129	400	382	324	465	406	360	380	494	538
4	950	1020	923	1005	1462	1194	933	1339	550	502	624	675	526	690	650	674	778
5	882	1140	715	1035	1522	1284	1053	1429	550	502	624	675	526	690	650	674	778
6	1032	1200	955	1125	1612	1344	1113	1489	550	502	624	675	526	690	650	674	778
7	1092	1200	1015	1125	1612	1344	1173	1519	550	502	624	675	526	690	650	674	778
8	1122	1080	1045	1004	1672	1434	1233	1549	730	562	864	825	676	960	860	854	898

	Progression of aerobic capacity – Speed (m/min)																	
	**AE**									**CE**								
Basal	30	35	30	25	30	30	15	15	25	25	25	35	30	30	15	15	15	20
4 Weeks	35	30	45	45	40	40	35	30	35	25	25	30	25	40	30	25	30	30
8 Weeks	50	45	45	40	45	50	30	40	30	35	35	40	35	35	40	35	35	30

	Progression of aerobic capacity – Distance (m)																	
	**AE**									**CE**								
Basal	396	532	549	310	459	456	480	96	325	360	310	637	480	429	108	108	97	182
4 Weeks	640	456	1143	1147	848	896	856	489	682	377	350	462	335	960	486	360	519	489
8 Weeks	1410	1215	1057	920	1080	1600	519	856	519	672	679	880	668	700	852	665	640	489

Individual data (manuscript)				
Reference – FIGURE 3				
Adipose tissue irisin levels (ng/µg of total protein content)				
RET				
CS	HS	AE	RE	CE
0.01866	0.00000	0.00766	0.01505	0.00744
0.01897	0.00000	0.01578	0.00258	0.01997
0.01717	0.00820	0.00349	0.11221	0.00531
0.00212	0.00157	0.01781	0.03000	0.01405
0.00167	0.00000	0.01835	0.03118	0.00690
0.00089	0.00000	0.01204		0.01255
	0.02049	0.00372		0.03331
MES				
0.01360	0.00000	0.07451	0.01456	0.02135
0.01729	0.00000	0.08111	0.07404	0.02527
0.01052	0.01574	0.04011	0.06234	0.06565
0.00458	0.01386	0.09285	0.04005	0.06110
0.00483	0.01083	0.01929	0.02975	0.02776
	0.07425	0.01781		0.02529
	0.05495	0.07739		0.03589
				0.09133
SUB				
0.00716	0.04340	0.03784	0.04399	0.04396
0.04054	0.03938	0.04309	0.04826	0.03692
0.00797	0.00722	0.03938	0.05038	0.00926
0.01027	0.03628	0.01288	0.06495	0.00925
0.00850	0.03830	0.03016	0.06286	0.03626
	0.01958	0.06406		0.07950
				0.05131
				0.04782

Individual data (manuscript)				
Reference - FIGURE 4				
Adipose tissue FGF21 levels (ng/µg of total protein content)				
RET				
**CS**	**HS**	**AE**	**RE**	**CE**
0.26963	0.24645	0.35263	0.50203	0.35544
0.25791	0.30981	0.45166	3.18788	0.28018
0.21057	0.31766	0.62753	5.42747	0.53670
0.27179	0.31090	0.48989	4.22827	0.51967
0.36310	0.65046	4.37365	3.51680	2.10014
0.42519	0.43794	3.59923		5.34196
		4.41755		3.07844
MES				
2.87107	4.83792	2.50440	1.93494	4.76329
3.20342	3.59017	2.10452	2.00928	1.24130
2.91981	8.07143	1.49518	2.33006	1.99098
2.86311	2.96380	1.83873	3.00060	1.72682
2.24978	8.33060	1.76459	2.99966	1.56030
2.05351	4.25275	1.54915	4.34107	1.44751
1.95967		1.47631	1.85062	2.76480
		3.11802		2.62317
SUB				
1.01495	0.57952	0.19118	0.51183	0.27836
0.11453	0.28257	0.61221	0.57055	0.68637
0.21583	0.47483	0.72229	2.36639	0.09257
0.24788	0.10683	0.64448	2.41846	0.12906
0.45208	0.36192	0.40227	2.83199	0.81326
0.12664	0.43953	0.86528	0.93236	2.82880
	0.20852	2.12809		1.65691
		1.64733		1.74780

Individual data (manuscript)				
**Reference - FIGURE 5**				
**Mesenteric adipose tissue protein content by Western blotting**				
**PGC1α**				
**CS**	**HS**	**AE**	**RE**	**CE**
65.56858	28.95870	122.43510	134.31190	104.58650
134.43140	142.65330	136.03560	248.37590	162.66440
132.86600	67.99791	213.06200	202.66430	103.21110
67.13400	83.56813	101.05420	148.17200	120.64700
58.80135		125.03640	140.08460	110.74700
100.00000				
**PPARγ**				
95.63132	81.50383	189.85480	142.12860	119.16310
99.96712	125.98950	95.89071	255.06040	81.07030
100.03290	24.87402	90.48955	111.97380	99.67004
64.68666	90.47100	93.33772	143.49980	140.34990
100.00000		92.08682		90.74310
		121.84660		
**UCP1**				
87.16769	49.35711	124.94850	94.43144	132.65830
112.83230	75.13518	86.33322	94.69455	86.91026
79.15746	65.10129	101.18200	69.84528	75.84942
120.84250	52.68858	146.31850	92.25698	83.24644
78.77081		83.07359	79.91333	90.81480
100.00000			105.66240	

Individual data (manuscript)				
Reference - FIGURE 6				
Subcutaneous adipose tissue protein content by Western blotting				
PGC1α				
CS	HS	AE	RE	CE
74.85400	71.67929	74.38078	119.86810	92.28613
72.58539	78.76723	70.33540	83.23515	82.76341
90.20462	66.44175	88.36640	143.04490	84.11970
109.79540	91.61154	114.16660	128.98870	97.82430
100.00000	90.85936	120.29210	107.01640	100.86630
	93.97688	91.02351	136.26360	112.45500
PPARγ				
80.57787	83.87445	83.99614	89.66069	83.11753
57.69430	83.66619	75.67892	152.84960	102.48120
88.32539	100.41470	84.97770	92.82798	95.21394
111.67460	88.50230	122.07290	106.14270	84.42376
100.00000	100.15680	96.29208	158.12760	95.66499
				107.95580
				110.41260
UCP1				
45.71877	23.81362	72.55727	131.83510	96.18973
154.28120	96.20131	123.04130	134.76830	90.60760
50.98295	74.17028	255.29780	96.87370	230.20680
88.15093	108.66760	135.64930	225.81100	204.87130
100.00000			241.96360	185.85060
